# Leverage structure decisions in Bangladesh: managers and investors’ view

**DOI:** 10.1016/j.heliyon.2021.e07341

**Published:** 2021-06-17

**Authors:** Mohammad Nazim Uddin

**Affiliations:** Department of Business Administration, International Islamic University Chittagong, Bangladesh

**Keywords:** Corporate governance, Determinants, Agency problem, Leverage structure decision, Bangladesh

## Abstract

The paper investigates the impact of managers' and investors' perceptions on financial leverage decisions in Bangladesh. To fulfill the purpose of the paper, the final structure of the questionnaire was made by adopting pre-testing and assessment of outer factor loadings and measures the internal consistency of all items in the test or scale using Cranach's Alpha. The composite reliability (CR) was tested by calculating the composite alpha and average variance extracted (AVE). The study employs partial least square structural equation modeling (PLS-SEM) to investigate the structured relationship between the observed and latent variables and extends the path analysis to test the hypotheses. The study reveals that corporate governance significantly and positively influences the leverage structure decision. The result intends to establish that if firms serve corporate governance, it will make the firms to manage more debt into the leverage structure decision. Results also reveal a negative and significant association between the determinants and financial leverage structure decision, and this relation signifies that when determinants tend to upturn, outside borrowing will fall into the financial leverage structure decision. The policy implications advanced from this study include the transformation of ownership structure, corporate governance, and financial policy to facilitate proper leverage structure decisions.

## 1 Introduction

Research on leverage structure decision had been vigorously emphasized, especially after the establishment of the first theoretical framework by Modigliani and Miller (1958, 1963) to address the topic from the firm's perspective. Despite extensive research, the leverage remains one of the controversial issues of modern corporate finance. An ongoing debate regarding a firm's optimal leverage decision presently exists. “How do firms select their leverage structure decision?” A three-decade old question of Myers (1984) remains unanswered. Multiple leverage structure theories have resulted in different hypotheses on leverage decisions. The Modigliani and Miller Theorem (1958) predicted that financial leverage structure decision is unrelated to firm value, while the trade-off theory explains that firm value is maximized by utilizing the optimum mix of financing (Myers, 1984). An optimal debt policy includes the least risk associated with cost of financing, by deriving the tax benefits aided to maximize the firm value.

No optimal leverage decision exists; therefore, the cost of deviation is observed to be negligible (Myers and Majluf, 1984). Many methods lead to different hypotheses regarding the independence of the leverage with firm value. There is no extensive empirical study on the financial leverage decisions in Bangladesh; however, researchers still observe differences in the results gathered, as evident from the study-to-study variations in the signs and statistical significance of the regression coefficient. According to empirical findings, the model which can better explain leverage decisions is unclear (Giudici and Paleari, 2000; Carpentier et al., 2007; Colombo and Grilli, 2007; Miguel and Pindado, 2001)).

The perceptions of managers and investors on determinants and corporate governance are vital for the optimal leverage decisions, which is usually influenced by the firm's capacity for leverage, tangibility, profitability, growth opportunity, liquidity, corporate tax, market interest rate, agency problem, and stock price (Baker, Ruback and Wurgler, 2004; Carpentier et al., 2007). The study focuses on managers and investors' views on such factors which will lead to financial leverage decisions. The proper evaluation of such elements can act as a trade-off between the agency problem and capital cost (Chittenden et al., 1996; Bevan and Danbolt, 2004). The decisions of corporate firms are supposed to make them behave differently from developed countries due to the presence of family and political interference in the management and financial market for over a decade. Firms are directed by political and family-affiliated individuals who acquire their license through political considerations (Uddin et al., 2019). Therefore, the concern in this paper is to examine the impact of determinants on leverage structure decisions from Bangladeshi managers and investors' perspectives.

Corporate governance provides a ground for enacting sound policies and wellness decisions. In over a decade, the aim, democracy and rule of law in Bangladesh became lost by the present government. The country lost its value, system, rules and good governance for improving the human index up to mark due to the corruption of its political leaders and the non-functioning of rules and regulations. The money and capital markets are dominated by a few families and politicians who influence firms' financial decisions and possess government support. Whereas, in developed countries, firms are managed by professional managers and dispersed shareholders who own corporate firms and are committed to complying with corporate ethics. Due to this, the minority shareholders were severely affected by the board of directors and CEO-duality (Jensen and Meckling, 1976), as managers had little influence firms' decisions making. Therefore, the study tries to examine managers and investors’ views on leverage structure decisions.

The following are some of the vital issues associated with the leverage structure decisions in Bangladesh:(i) Firms are regulated by families and politically affiliated individuals who are inexperienced in firms' operation and decision making(Rahman, and Rana, 2018). (ii) Managers possess limited authority in the implementation of policies and decisions, and are not independent in their positions. Consequently, they are being financially exploited by owners, resulting in the agency problem. Such corporate state further limits the scope of making optimal financing decisions (Habib, 2019). (iii) Few people and institutions who, under the support of the government, have dominated the money and capital markets where they manipulate billions of dollars from the banks and stock markets (Rahman, and Rana, 2018; Rashid, and Johara, 2018; Habib, 2019). Therefore, clients and investors become hopeless and discouraged to involve in the financial system. In such an environment, how does a firm obtain financing from the financial system? Therefore, this paper attempts to examine the impact of corporate governance on leverage decision from the managers and investors’ views.

Unlike previous studies, the paper contributes to the current literature in the following ways: (i) we provide a new test results on leverage decisions based on managers and investors' perception to make optimal financial leverage decision by mitigating the agency problems, because managers had gained professional experience and field information regarding the firms' operations. This study is the first empirical research to investigate managers and investors' views on financial leverage structure decisions.(ii)The previous studies were investigated by the secondary data published in corporate annual reports, which may not only explain the financial leverage decision in a firm, but also consider the behavioral finance such as ownership behavior, manager behavior and political regulations. In contrast, this study only focuses on managers and investors' views to explore their expectations on leverage structure decision, as their behaviors can influence decision making. Bajaj et al. (2020) focused on the great scope of primary research on capital structure decision as they could not identify any primary research despite investigating a long literature review for last 21 years. (iii) Most of the firms are managed by families and politically connected persons who are getting the support of government for which corporate governance is badly compromised. This occurrence has already crumbled the money and capital market in Bangladesh, and links to financial leverage structure decisions. Such corporate culture might further even collapse the industry on a global scale.(iv) The study tries to identify the main reasons of agency conflicts or unhappiness of managers associated with working commitments, which might influence the financial leverage decision. The agency problem results in huge managerial costs which subsequently reduces the firm value. The nature of agency problem could be understood from the shareholders and managers’ views, which is crucial in establishing leverage structure decision. (v) The study tries to investigate that how bad corporate governance practices abolish the financial theories and policies in developing countries like Bangladesh. In such a situation, this study is relevant, as it provides a guide for the government, policymakers and regulatory bodies to sustain the economy.

Although past studies had examined the factors worth considering for leverage decisions (Miller, 1988),they were limited to investigating the specific factors for an optimal leverage decision and did not focus on addressing the following questions:(i) why do investors and managers desire an optimal leverage structure decision for the firms? (ii) What are the main constraints of the optimal leverage decisions made by the corporate firms in Bangladesh through the adoption of financial polices? In the process of answering two questions, this study examines the investors and managers' perceptions on the leverage structure decisions in Bangladesh, owing to the non-optimality, but rather simplistic and heuristic nature of the formulated leverage structure decisions by Bangladeshi firms, which disregard the views of managers and shareholders. This study has been structured as follows: Part 2 captures the capital structure theories, related literature and framework; part 3 includes the sample, data collection, and methodology; part 4 imparts the interpretation of findings; and part 5 draws the conclusion and policy implications.

## 2 Literature review

The capital structure theories and empirical studies were critically investigated and divided into several parts:

### 2.1 Theories of capital structure

**The Modigliani-Miller Theorem (1958)** was the first to introduce hypothesis on capital structure decisions. The theorem opined that the capital structure was unrelated to the firm value under some realistic assumptions. The theory's assumptions were of a perfectly competitive market, absence of tax, inflation and transaction costs, and no information asymmetry. These assumptions are unrealistic in the modern economy, as it was impossible to find a single country ignoring corporate tax, transaction costs, information asymmetry, and inflation simultaneously. As a result, no basis has been developed by the theory on the implementation of capital structure decision, which is extensively criticized. Besides, it was also predicted that information was symmetrical due to companies' confidential disclosure (Hamada, 1969; Hatfield et al., 1994; Stiglitz and Weiss, 1981). Later, the Modigliani and Miller (1963) modified their theory, with firms maximizing their firm values by employing more leverage in their capital structure due to tax shields. Kopecky et al. (2018) utilized an alternative capital structure policy which was deviant from the Modigliani and Miller (1958) and Miller (1988) theorem for the prediction of borrowing funds that reinstates the irrelevance of the capital structure. **The Trade-Off Theory**: An investigation on the use of the capital structure theories in 227 Vietnamese listed firms indicated that the trade-off theory was adopted to explain their implemented financial decisions (Nguyen et al., 2019). According to Bajaj et al. (2020), a total of 183 research papers were analyzed on the capital structure theories in developing countries, but a lack of primary research still exists. This review indicates the dominance of the trade-off theory in financial decisions. In contrast, the trade-off theory was developed from the Modigliani and Miller (1958) debate, which highlighted that the capital structure was unrelated to firm value. If corporate tax was introduced with the Modigliani and Miller's proposition, then, the benefits of debt will be derived. Cheng et al. (2010) revealed that the trade-off theory was developed by Modigliani and Miller (1963), and that the capital mix could be maximized from the firm value by the trade-off between tax benefits and the cost of debts. According to the trade-off theory, a firm combines its debt with equity when the benefits of tax due to the use of debt are equal to the bankruptcy cost, resulting in the firm value attaining an optimal level. **Pecking Order Theory**: This is widely used to analyze the financial behavior of corporate firms and has a set and detailed optimal leverage ratio (Myers, 1984).According to this theory; companies are expected to select their capital structure from internal to external sources. Donaldson and Davis (1994) indicated that firms with strong internal cash flows utilize their first internal source before proceeding to an external capital, while Myers and Majluf (1984) opposed to generating an external finance prior to the exhaustion of retained earnings, as debt capital should only be considered as a last resort (Arnold, 2008). Razak and Rosli (2014) investigated the capital structure theories by considering 200 listed firms in Malaysia from 2007 to 2012.The findings reveal that the trade-off and pecking order theories are explained by the financial decisions, as firms follow the pecking order theory by continuously investing in internal funds due to sustainment and growth. Indeed, gearing ratios create an imbalance between cash flow, dividends and investment opportunities, and are resorted to when firms require external funds. Myers (2001) opined that firms should employ the pecking order theory when sufficient internal cash flows are generated. **The Market Timing Theory**: The theory was introduced by Baker and Wurgler (2002), and maintains that a company should only issue shares when the market is favorable, and in the event that the stock price declines, it should proceed to repurchase the stock by debt. This process of financing is on behalf of shareholders' wealth maximization. Baker and Wurgler (2002) exposed the positive impact of market timing on the firm value of US companies. The theory lays down some assumptions such as the adjustment of stock market messages, which is in line with the implementation of financial decisions. Therefore, compared to other theories, the Market Timing Theory better addresses the goal of corporations. **The Signaling Theory**: This refers to the information discrepancy existent between managers and shareholders. In this context, managers are expected to issue shares when overvalued and give bonds when undervalued, in a bid to deriving the benefits of information asymmetry (Ross, 1977: 23). This capital market environment is marked as an information differentiation between managers and shareholders which will lead to a reduction in bankruptcy. **The Agency Cost Theory**: The causes of conflicts and agency costs are from the separation of ownership from management, different risk preferences, information asymmetry and moral hazards. Another agency problem is created between lenders and managers or owners when debt capital is incurred (Panda and Leepsa, 2017; Jensen and Meckling, 1976). Titman and Wessel (1988) suggested that costs resulting from agency problems might be more severe for companies (Chung, 1993), while Jensen and Meckling (1976) revealed that agency problems might even be created to use debts.

### 2.2 Empirical evidence and hypothesis

Three dimensions of relationship exist in leverage structure decisions and previous studies have reviewed these viewpoints as follows:

#### 2.2.1 Determinants of leverage structure

Determinants can be defined as the issues or factors that a firm takes into account, when making its financial leverage decisions. A firm always desires to use the maximum level of debt in its capital structure depending on such factors. The determinants of leverage structure on 34 Australian listed property companies were studied. This empirical evidence provided that profitability, growth opportunity, operational risk, and size influenced the leverage structure of the firm(Chikolwa, 2014).The 972 listed Chinese companies considered profitability, operational risk, age, ownership structure and corporate governance to determine the debt behavior of the firms(Chen, and Strange, 2005).The capital structure decision depends on various factors, such as that highlighted by Sibindi (2016),where he opined that the leverage level of firms depends on a tax shield. If the firms exist in a high tax bracket, they tend to utilize their higher levels of debt to pursue the maximum tax benefit for increasing the firm value (Sibindi, 2016; Arvanitis et al., 2012). Hoque et al. (2014) emphasized that debt was determined by the factors of financial risk, profitability, availability of funds, productivity, liquidity, operating risks and corporate tax, and also independent variables such as capital structure, debt-to-equity ratio, debt-to-asset ratio and fixed asset to total assets have significant influence (up to 79.1 percent) on the value of the firm. Husain et al. (2012) observed that profitability, tangibility, liquidity and managerial ownership had significant and negative impact on leverage, in corroboration with the results of (Alom, 2013). A positive and significant impact of growth opportunity and non-debt tax shield on leverage was also observed (Siddiqui, 2012; Jahan, 2014). Sayeed (2011) presented the total debt to the market value of the company as the leverage ratio in one equation, and the long term debt to market value in another equation. Lima (2009) observed that the growth rate, operating leverage, tangibility and debt service capacity were positively related to the capital structure; while contrastingly, the agency cost of equity and bankruptcy risk negatively affected the debt ratio. The leverage structure is positively influenced by determinants such as firm size, corporate tax, growth, profitability and tangibility, investment opportunity, and stock price volatility(Hasan et al., 2014; Hossain and Hossain, 2015).Unfortunately, there is a scarcity of evidence in discussions concerning capital structure theories in the aforementioned literature. Therefore, the following hypothesis proposed:name.

*H*_*1*_: Determinants can positively influence the leverage structure decision

#### 2.2.2 Corporate governance and capital structure decisions

Corporate governance plays a key role in any decision making, owing to the positive influence of corporate ethics, values, and principles on corporate decisions. The use of corporate principles will help a corporate firm to receive more borrowings. One of the leading factors influencing the leverage structure decisions is corporate governance that affects the financial and strategic decisions of firms (Adusei and Obeng, 2019; Detthamrong et al., 2017) The board size and board independence are more positive, and the impact of CEO-duality is negatively significant for the financing decisions of the firms (Zaid et al., 2020).The board size, board composition, and CEO-duality negatively influenced capital structure decisions (Bansal and Sharma, 2016). On the other hand, Setayesh et al. (2012) exposed that the ownership concentration, percentage of non-duty members of the board, and board independence have no significant effects on capital structure (Achchuthan et al., 2013). Strebulaev (2007) indicated that in the presence of frictions, firms adjust their capital structure infrequently. Jaradat (2015) found significant impact of board size, board gender, external directors and CEO-duality on the capital structure decisions from 2009 to 2013. Sueyoshi et al. (2010) indicate no significant impact of board size on firm performance. However, some researchers argue that a large board size can make the board liberty and diversity, which is beneficial to enhance the firm performance (Ciftci et al., 2019). Rehman et al. (2010) find a positive and significant relationship between the board size and the capital structure decision. It was discovered that such corporate firms focused on corporate governance was always preferred to receive external debt. The professional managers and industry experts, in contrast to private owners, might be beneficial to the firms in terms of knowledge, skill, and experience in solving the problems of market, financial, and board monitoring(Aldatmaz and Brown, 2020; Meuleman et al., 2014).Therefore, hypothesis *H*_2_ is stated thus: a positive relationship exists between corporate governance and leverage structure decision.

#### 2.2.3 Leverage structure decisions

Till recently, both theories, trade-off theory and pecking order theory, are employed to explain leverage structure decisions of firms. Previous studies were investigated to show the effects of both theories separately on capital structure decisions (Warmana et al., 2020). The choice of leverage structure is an important financial decision of a firm, since the impact of capital structure on the profitability is significant. Several studies provide a positive relationship between the use of debt and profits. Although companies were yet to employ any ideal leverage ratio, this indicator could be moved in specific contexts (Hussain et al., 2021). Several studies advanced in the developed countries focusing on the relationship between corporate governance and leverage decision using the samples of developed countries. As a new emerging country, very few investigations are conducted by china on the important topics of corporate governance. It seems that there is a strong relationship between corporate governance and leverage structure (Liu et al., 2012; Ho et al., 2011). Morellec et al. (2012) revealed that agency problems could better justify corporate financing decisions, as the manager is capable of issuing less or more debt to derive personal benefits. Leverage decision relies on the ways of how corporate governance is maintained by the firms using the sample of non-financial listed firms in China during 2000–2018. Empirical results show that better quality of corporate governance negatively influence the financial leverage decisions of the firm. Governance had an abundant effect on capital structure decision, which included effective control, accounting system, better management, skills and monitoring as well (Zhou et al., 2021; Ganiyu and Abiodun, 2012). Haque (1989) investigated a study on Bangladeshi firms which establishes that the capital structure depends on the nature of the industry, and no significant impact of capital structure on a firm's profitability, dividend, and market value exists (see Tables 1 and 2).

**Table 1:** The Methodologies and data source of previous studies (2010–2020).

Literature Types	Results	Data	Methods	Previous Studies
Capital Structure Theories	Firm size, profitability, tangibility, and Fund flow are significant. Razak and Rosli (2014) found that NDTS, firm size and growth influenced the capital structure.	Secondary	GMM, Linear Regression Analysis	Nguyen et al. (2019), Razak and Rosli (2014), Cheng et al. (2010)
	M&M equilibrium is not unique, but this theory should be a key issue for leverage decision for financial officers, and creditors. Myers (2001) says that capital structural theories are not universal and focused on the mix of debt-equity depending on tax shield.	Secondary Research	Conceptual Analysis	Kopecky et al. (2018); Sibindi (2016); Arvanitis (2012).
	Literatures on capital structure theories for the last 21 years were studied. Most studies are focused on developed counties, but there was no primary research in the whole study.	Secondary Research	Conceptual Analysis	Bajaj et al. (2020)
	Market-book value ratio negatively influences the capital structure.	Secondary Research	OLS regression	Baker and Wurgler (2002); Setyawan (2011).
Determinants	Profitability, collateral, liquidity, market to book value, tangibility, size. Financial risk, growth, operating risks and corporate tax. Agency cost, non-debt tax shield, growth opportunity, nature of assets,	Secondary Research	OLS regression, FGLS, Fixed effects,	Alom (2013).; Arvanitis et al. (2012); Jahan (2014); Siddiqui (2012). Hoque et al. (2014); Sayeed (2011).
Corporate Governance	Board size, board composition, CEO duality, audit committee, ownership concentration, institutional ownership, Board gender, outside director, Board Committee. The board size and board independence are more positive, and the impact of CEO-duality is negatively significant for the financing decisions of the firms	Secondary Research	Multiple regression, Fixed effects,OLS, GMM.	Bansal and Sharma (2016); Setayesh et al. (2012); (Achchuthan et al. (2013); Uddin et al. (2019); Zaid et al. (2020); Aldatmaz and Brown (2020); Meuleman et al. (2014).
Behavior, and Social science Research.	In the corporate, social behavior, or qualitative issues, PLS-SEM is considered to be a more appropriate tool for advanced research, and it has gained popularity in behavior and social science research comprising factor analysis	Primary Research	Outer loading factors, Cranach's alpha, PLS-SEM.	(Hair et al., 2014; Richter et al., 2015; Ali et al. 2019a, 2019b; Cheah et al., 2019; Hair et al. 2014, 2014; Chin and Newsted, 1999).

**Table 2:** Sector wise sample distribution.

No	Nature of Industry	Samples	Population	% of the Population
01	Jute & Textile Sector	11	52	21
02	Food and Allied Sector	8	18	44
03	Engineering Sector	9	36	25
04	Tannery, Footwear and Fuel & Energy Sector	6	24	25
05	Pharmaceutical & Chemical Sector	12	28	43
06	Cement & Ceramic Sector	7	12	58
07	IT and Telecommunication	5	10	50
08	Miscellaneous	5	14	36
09	Total	n = 63	N = 194	33

Nevertheless, still now, no study has been done to include the impact of managers' and investors' view on leverage structure decisions (Bajaj et al., 2020). Therefore, the inclusion of such view might lead to an optimal leverage structure decision for the firms by reducing agency problems and default risks as the inclusion of managers and investors' view will focus on the good intention of stakeholders, as well as firms’ ability (Zhou et al. (2021)).This study bears very novelty in scopes, methods and results to cover the gap of previous research. Based on the gap of previous studies, the study focused on qualitative factors of determinants and corporate governance link to leverage structure decisions by using the perceptions of decision makers, thereby applied the suitable method coincides the nature of data. However, this study will bear an enormous value on existing literature due to it is the first and novel study on the perception analysis of managers and investors. Without allowing the perceptions of managers and investors, no optimal capital structure decision will be formulated.

## 3 Conceptual frame work development

**Figure 1:**
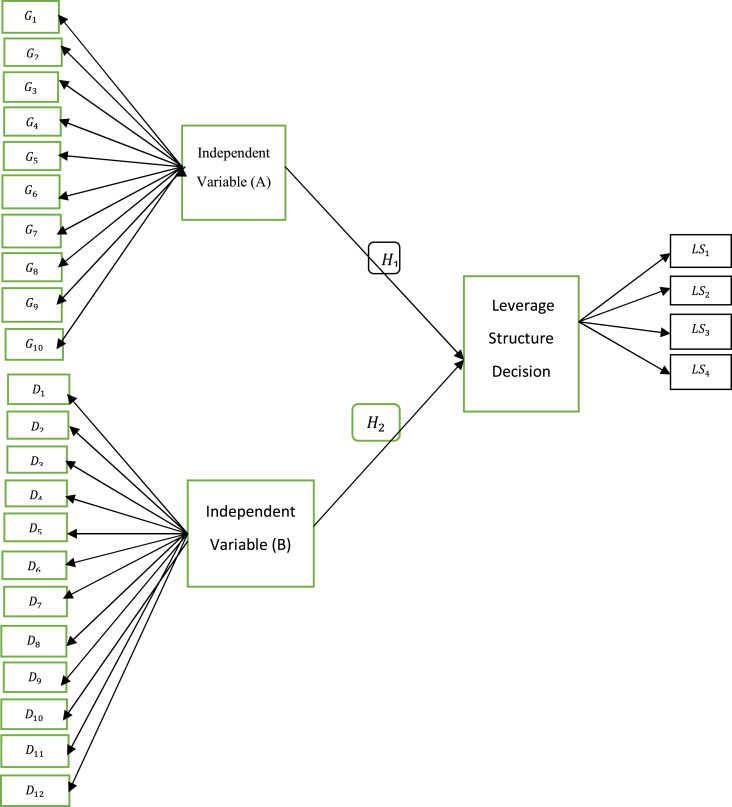
Framework.

**Figure 2:**
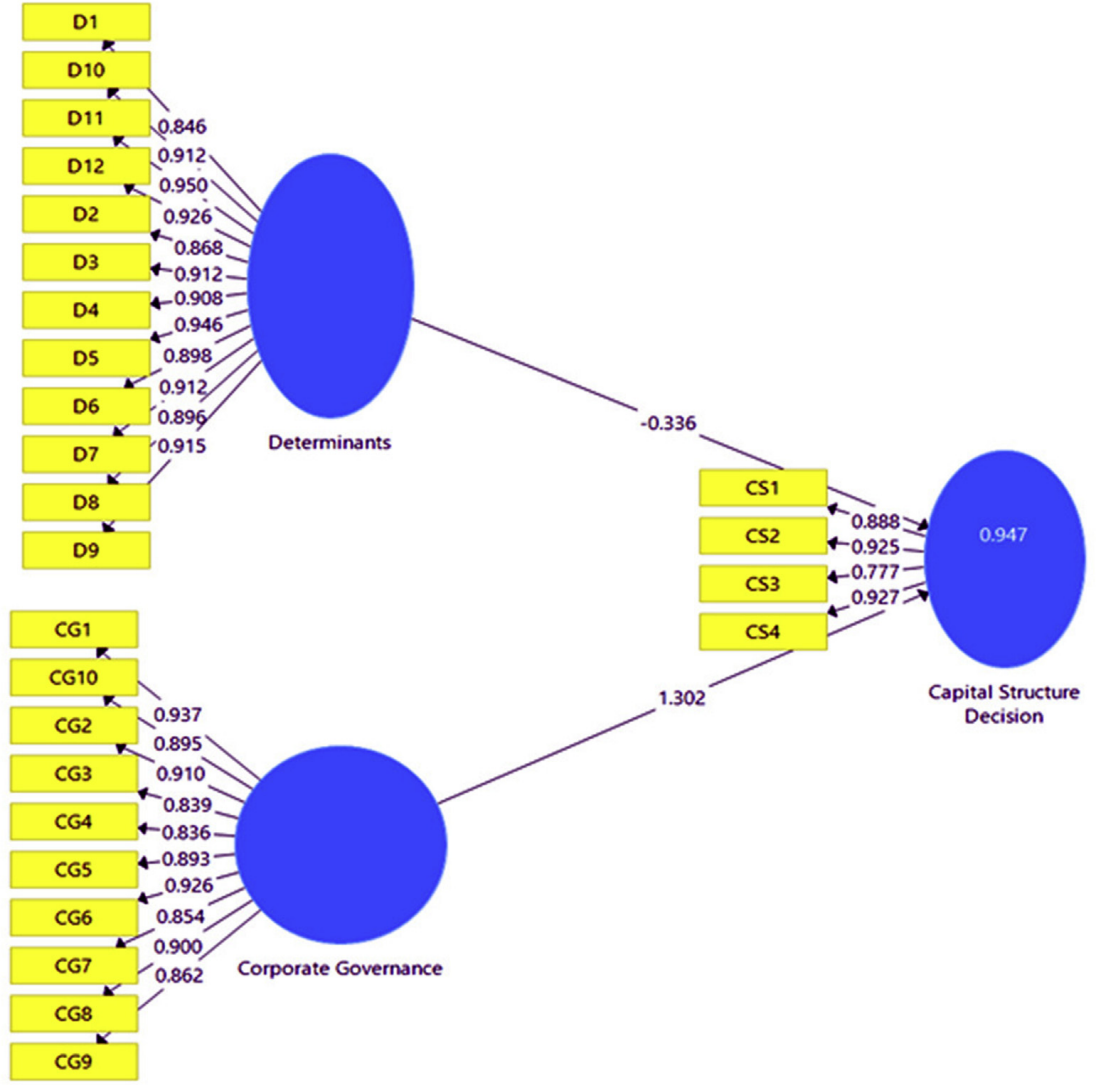
The structural equation model for the leverage structure decision.

The conceptual framework depicts how corporate governance, as well as determinants influences the leverage structure decision exhibited in Figure 1. This study is to investigate the impact of determinants, and corporate governance on financial leverage decision of corporate firms in Bangladesh. In this purpose, the determinants and factors of corporate governance are chosen by the use of previous studies, industrial experts and pilot survey, which may be involved with financial leverage decision (see Figure 2).

The conceptual link provides a structural relationship between corporate governance, determinants, and leverage decision. In such structural relationship, there are 10 factors of corporate governance plays an important role to evaluate the corporate governance that will influence the leverage structure decision, which is regarded as first independent variable. Whereas, there are another 12 factors take part in total determinants of leverage structure to decide the leverage structure decision, which is considered a second independent variable. Therefore, the study is an initial effort to examine the joint impact of such corporate governance, and determinants on financial leverage decisions of corporate firms in Bangladesh.

### 3.1 Sample design

Of the 338 companies listed on the Dhaka Stock Exchange, 136 are financial institutions (banks and non-banks), while eight are real estate, travel and leisure companies. The study excluded full financial sectors, some real estate companies, and travel and leisure companies from its investigations. The financial sector was excluded in the study for its variation in leverage structure and regulations from the manufacturing sector because it had minimum regulatory capital requirement (Diamond and Rajan, 2000). The real estate and leisure companies were not considered due to the long-term postponement of their trading activities on the DSE. However, considering the various encountered limitations, the following companies were selected for investigation to fulfill the purpose of the study:

Ultimately the study had chosen 63 companies out of 194 companies purposively. Out of sixty three companies 21% are from jute and textile companies because of its financial ambiguity and 44 % are from food, and allied sector, the sector was small. 25% are chosen from engineering as it limits publicly financial disclosure, and 25% are from tannery, footwear and fuel and energy sector, the reason for small size was a limit of availability in the public. 43% are from pharmaceutical and chemical sector; 58% are selected from cement and ceramic sector, and 50% are from IT and Telecommunication, as these sectors are the finest in the macro-economy. Finally, 36% are from miscellaneous sector, which are scatter in the industry. However, the aforementioned samples are considered by the study basing on the availability and easy mode of collection to the concerned.

### 3.2 Data collection

The research is designed basing on previous literature, which didn't contain any psychological issue or primary works on financial leverage decision or debt policy. Most of financial decisions rely on stock market situation, corporate governance, and technical skills of managers the study was primarily responsible to explore such impact on. Bajaj et al. (2020) investigated the literature of 21 years on capital structure theories could not explore any primary research, and gave a direction that there is a great scope of doing research on primary data. Questionnaires were employed as the research instrument for data collection and comprised of two sub-units for managers and investors' views of companies listed on the DSE in Bangladesh. The questionnaire included issues of objectives, determinants, corporate governance and leverage structure decisions. The first part contained six questions capturing the respondents' profiles, and another eight for the sample companies to justify their data reliability and acceptability. The second part of the questionnaire consisted of both determinants and factors of corporate governance for leverage structure decisions. Managers' and investors' perception were measured using 5-point Likert-scale ranging from 1 to 5 indicates from strongly disagree to strongly agree as in the study of Craig and Dibrell (2006). The questionnaire involves two sub-sets: one set is available to the financial managers or chief financial officers (63), while the other is available to the key shareholders (126) to explore their views on leverage structure decisions. Responses were received from sixty-three financial managers, and one hundred and twenty-six investors, by allocating a month or more. The respondents' individual information is analyzed below for validity and reliability of data:

Table 3 shows the demographical profile of participants that a total of 189 samples were chosen for the investigation of perceptions of managers and investors, as their perceptions have an important impact on the leverage structure decisions. In this investigation, out of total 189 participants, 78% were male, and 22% were female participants. 18 percent are from the age group between 26 and 35 years. 28% are from the age group 36–45 years, and 30% of sample is from the age group between 46 and 55 years. 24% participants are the age between 56 and 65 years. Moreover, 91% are got married, and 9% are single. The educational position indicates that 2% hold graduation; 83% obtained post-graduation, and 15% have completed professional degrees. In terms of work experience, 5% are from 5-10 years; 66% have gained between 16 and 20 years; and 15% have acquired experiences between 21 and 25 years.

**Table 3:** Demographic statistics of respondents.

Sample Size: 189		Frequency	Percentage
Sex	Male	147	78
	Female	42	22
Age	26–35	33	18
	36–45	53	28
	46–55	57	30
	56–65	46	24
Marital Status	Single	17	9
	Married	172	91
Academic position	Graduation	5	2
	Post-Graduation	156	83
	CA/CMA/FCA	28	15
Experience	5–10	10	5
	11–15	11	6
	16–20	124	66
	21–25	28	15
	26–30	16	8

Table 4 reveals the leverage decision holders of the corporate firms. According to the results of investigation, it was clear that 81% leverage structure decisions are by the board of directors; and 19% are made by the chief executive officer (CEO) in the firms. However, none of the chief financial officers or financial managers participated in the leverage structure decisions. The main fact is that firms are led by a few families or government-backed politicians, who play significant role on the board for leverage structure decisions, and chief-financial officers, just carry out their orders in the firms. Professional knowledge and experiences remain unutilized in the firms, resulting to agency problems, which is opposite to optimality in decision.

**Table 4:** Leverage structure decision level of firms.

Decision Makers	Decision	
	Frequency	Percent
Board of Directors	51	81
Chief Executive officer	12	19
Chief Financial officer	0	0
Total	63	100

### 3.3 Material and methods

The review of previous literature and local corporate culture indicate that both determinants and corporate governance influence the financial leverage decisions. The contents of questionnaire were examined by 10 financial managers and 15 investors who suggested us for the exclusion of unnecessary contents in the questionnaire for final investigation. Subsequently, we conducted a pre-test by the help of professionals and practitioners before full investigation, who recommended the removal and addition of some items to improve the questionnaire. According to Urbina (2014), a pre-test is conducted by professionals or practitioners before a full investigation, to ensure the quality of questions. However, the final structure of the questionnaire was prepared by the pre-testing and testing of the outer factor loadings, and in order to serve the purpose of the study, it was necessary to measure the internal consistency of all items in a test or scale to reveal the validity of the questionnaire data, which was primarily investigated by a test case of 50 responses using the Cranach's alpha. All the items in this test were found to be in correlation with one other. This is because each of the alpha values exceeds the standard value. The alpha is a vital concept in the evaluation of questionnaire items and is often used to ascertain the validity and accuracy of an investigation. Sequel to ensuring the accuracy of the results of the pilot survey using the Cronbach's alpha, the composite reliability test was conducted on the overall sample items to assess its reliability, which revealed significant level of satisfaction, given the obtained values of the composite alpha and AVE. Afterwards, the convergent validity was assessed to determine the unique correlation among the constructs in this scale. In order to assess the discriminant validity, the process suggested by Fornell and Larcker (1981) was applied. Subsequently, the study applied the structural equation model to assess the structured relationship between the observed and latent variables. For questionnaire variables or behavioral analysis, SEM is more useful to detect the joint impact of independent variables on dependent variable. Under the SEM, the path analysis was used to assess the research hypotheses, which reveals the direct, indirect or total effects of each independent variables on the dependent variable (Leverage structure decision), so that the correlation between the independent variables and dependent variable could be logically explained. The path analysis aims to interpret the quantitative estimate of the causal links among a set of variables. The SEM is a more efficient and convenient multivariate analysis to investigate the structured relationship between the observed variable and latent variable, and it has gained popularity in behavior and social science research comprising factor analysis. The PLS-SEM is given priority over the CB-SEM, as it is considered to be a more appropriate tool for advanced research (Hair et al., 2014; Chin and Marcoulides, 1998; Malhotra et al., 2006; Richter et al., 2015; Ali et al. 2019a, 2019b; Cheah et al., 2019; Hair et al. 2014, 2014; Chin and Newsted, 1999). Therefore, in path analysis, the theoretical framework is investigated based on the model of the causal links between variables, through conversion into an empirical model of research.

## 4 Empirical results and discussion

In this section, the results of factor analyses such as the Cronbach's Alpha, composite reliability for measuring reliability, validity of instrumental variables, as well as correlations among observed variables are analyzed. The impact of the perceived variables on leverage structure decisions are investigated by structural equations, while hypotheses are established by the path analysis.

### 4.1 Results of the reflective measurement model

Table 5 presents the status of each questionnaire variable to assess its contribution to its assigned construct. An outer loading is an initial test conducted to identify the individual contribution of questionnaire constructs to its assigned constructs. All outer loading factors have established that the individual contribution of the constructs had attained the cut-off points of 0.70 or greater, which provides the greatest reliability of the study. The results of factor loadings have received the criteria, as recommended by Hair et al. (2014). The values found lower than 0.70 may be discarded from the observed variables of the research instruments, in which outer factor loadings were the guiding principles for selecting the contributed variables in the study (Hulland and Ivey, 1999). Therefore, this manner of selecting the contributed variables on the research instruments is valid in explaining the structured relationship existent between the observed variable and latent variable in this study.

**Table 5:** Outer loadings of the measurement model.

SL	Details of Questionnaire items	Factors	p-values
**Issues of Corporate Governance for Leverage Structure Decisions**			**1**
$CG_{1}$	The rights of shareholders are recognized by your firm	0.937	0.000
$CG_{2}$	Board Size and composition have an influence on leverage decisions	0.910	0.000
$CG_{3}$	Corporate laws and regulations are observed by your firm	0.839	0.000
$CG_{4}$	Independent directors are on board (at least 10%)	0.836	0.000
$CG_{5}$	The rights of stakeholders are protected by law.	0.893	0.000
$CG_{6}$	Financial reports are timely prepared and delivered to stakeholders	0.926	0.000
$CG_{7}$	Firms can freely appoint auditors	0.854	0.000
$CG_{8}$	Your firm provides fairly, timely and cost effective information	0.900	0.000
$CG_{9}$	Annual general meeting(AGM) is regularly held	0.862	0.000
$CG_{10}$	There is female board member participation	0.895	0.000
**Leverage Structure Decision**			**2.0**
$LS_{1}$	Issuing common stock/external equity financing	0.888	0.000
$LS_{2}$	Issuing bond/debenture/Long-term loan	0.925	0.000
$LS_{3}$	Issuing preferred stock (redeemable/Perpetual)	0.777	0.000
$LS_{4}$	Retained Earnings/Internal equity financing	0.927	0.000
**Determinants of leverage structure Decision**			**3.0**
$D_{1}$	Profitability results in leverage decision	0.846	0.000
$D_{2}$	Growth opportunity influences the leverage decision	0.868	0.000
$D_{3}$	Tangibility has an impact on leverage structure	0.912	0.000
$D_{4}$	Changes in market interest rates influence leverage decision	0.908	0.000
$D_{5}$	Corporate tax rates directly involve leverage decision	0.946	0.000
$D_{6}$	Business risks influence leverage decisions	0.898	0.000
$D_{7}$	Agency cost is related to leverage decision	0.912	0.000
$D_{8}$	Liquidity has an impact on leverage decision	0.896	0.000
$D_{9}$	Investment opportunities directly influence leverage decision	0.915	0.000
$D_{10}$	The firm size can influence leverage decision	0.912	0.000
$D_{11}$	The nature of industry influences leverage decision	0.95	0.000
$D_{12}$	Stock price volatility influences leverage decision	0.926	0.000

Source: The results were estimated by the researcher using questionnaires.

CG = Corporate governance; LS = Leverage Structure; D = Determinants.

The Cronbach's Alpha for leverage structure (0.902), corporate governance (0.969) and determinants (0.981) all exceed the standard value of 0.70, indicating that the level of reliability for investigation is satisfactory as suggested by Tavakol and Dennick (2011). Thus, the coefficients received from all queries in the Likert scale are trustworthy. The composite reliability is preferred over the Cronbach's alpha, as it measures the overall reliability of the investigation, and it is believed that the coefficients derived from all questions in the Likert scale are reliable and acceptable. The CR and AVE are used for the evaluation of the convergent validity, as suggested by Hair et al. (2010). The convergent validity refers to those responses on an instrument showing a strong relationship with responses from similar devices. Higher values of the CR and AVE provide an avenue for objects in establishing meaningful inferences from a phenomenon. Table 5 reveals that the CR and AVE values exceed the standard value of 0.70 and 0.50 respectively, as suggested by Hair et al. (2014). Therefore, the inferences on the instrumental variables are more reliable and acceptable in investigation of the structured relationship existent between the independent and dependent variables.

Table 7 presents the discriminant validity of our investigation, which was evaluated using the Fornell-Larcker Criterion. The discriminant validity recommends that strong correlations amongst each other should be avoided by all constructs in an instrument. No standard scale exists for the assessment of acceptance level to assess the multicollinearity problems among the constructs. Discriminant validity is used as an appropriate method to prevent the multicollinearity issues.Table 6 indicates that all constructs in the designed instrument have attained a satisfactory discriminant validity result, owing to the direct proportionality of the square root of the average variance extraction (diagonal) and the correlations (off-diagonal) for all constructs.

**Table 6:** Construct reliability and validity.

Items	Cronbach'sAlpha	Composite Reliability	Average Variance Extracted(AVE)
Leverage Structure	0.902	0.933	0.776
Corporate Governance	0.969	0.973	0.785
Determinants	0.981	0.983	0.824

Source: The results were estimated by the researcher using questionnaires.

The cross-loadings were used to determine the discriminant validity by investigating the cross-loadings of the indicators. The results, as depicted in Table 7, indicate acceptable discriminant validity, since the results of outer loadings on the involved construct exceed all of its loadings on other constructs. Each indicator requires having a higher loading on its construct but low loadings on the other construct (Hair et al., 2014). This implies that the correlation of similar objects exceed other objects, and provides a lesser correlation between two independent variables. The discriminant validity is investigated by matching the cross-loadings amongst each other. The cross loadings is used to measure more than one significant factor, which can help to check the absence of high multicollinearity. Each item requiring a high-level loading on its construct, but a low level on other constructs does not imply the existence of any multicollinearity problem. Table 7 indicates that all items in the questionnaire gave higher constructs than the others, confirming the acceptance of the discriminant validity.

**Table 7:** Discriminant validity using the fornell-larcker criterion.

	Leverage Decision	Corporate Governance	Determinants
Leverage Decision	0.881		
Corporate Governance	0.971	0.886	
Determinants	0.949	0.986	0.908

Source: The results were estimated by the researcher using questionnaires.

### 4.2 Results of the structural equation model

The model aims to estimate the structured relationship existent between objects or theoretical constructs as a mix of factor analysis and path, or regression analysis. A framework can be drawn by developing a theoretical relationship between the observed and latent variables. The following structural equation provides a triangular relationship between corporate governance and capital structure, or determinants and capital structure decision.

Table 8 presents the structural equation model results by showing the structured relationship between the independent variables (determinants and corporate governance) and dependent variables (leverage structure decision). The study reveals that corporate governance positively influences the leverage structure decision to a large extent. The use of outside borrowing depends on the implementation of corporate governance rules, as fair corporate governance tends to promote the faith of borrowers in corporate firms and incur more debt into their leverage structure. Otherwise, firms will lose in their bid to increase external debts in the leverage structure due to bad governance. Currently, there is a collapse in the corporate governance in Bangladesh due to the manipulation of the money and stock market by few families and politicians having connections with the government. Corporate governance had seriously declined for more than one decade with the collapse of the financial market under the prejudicial roles of regulatory bodies, and government. Apart from the role of regulatory bodies, the corporate firms are operated by a few families or persons, who are being helped by political governments(Uddin et al., 2019; Rahman, and Rana, 2018; Rashid, and Johara, 2018; Habib, 2019). The results of the SEM reveal it alignment with the trade-off and agency cost theories. According to the trade-off theory, a firm can extend its creditworthiness for external borrowing by exhibiting corporate governance attributes in the financial market (Cheng et al., 2010), while an agency problem is visible from weak corporate governance and will mitigate a firm's ability to include debt in its leverage structure. Therefore, the hypothesis on the relationship existent between corporate governance and financial leverage structure decision is subsequently established (β = 1.302, and p-value = 0.000). Whereas, the result also indicates that the aggregate impact of the determinants on the leverage structure decision is negative and significant, indicating that when determinants worsen, a rise in the debt in the leverage structure will be experienced. If the size of the determinants is increased by 1 percent, the leverage will be adversely affected by 0.336 units. The results reveal an opposite from the previous studies and the trade-off theory due to the unstructured government and political philosophy. Most of the firms are managed by a few families and politicians who are connected to the government, and can use their influence to manipulate their interests within the firms (Rahman, and Rana, 2018; Rashid, and Johara, 2018; Habib, 2019). The impact of the government and politics on the banks and stock market negatively influences the logical and systematic factors in Bangladesh, for which determinants differ from previous studies and theory (β = -0.336, and p-value = 0.000) (Rahman, and Rana, 2018). Therefore, the hypothesis on the positive relationship existent between the determinants and leverage structure decision is hence rejected (see Table 9).

**Table 8:** Cross-loadings.

	Capital Structure Decision	Corporate Governance	Determinants
LS1	0.888	0.803	0.767
LS2	0.925	0.892	0.877
LS3	0.777	0.832	0.847
LS4	0.927	0.89	0.846
CG1	0.903	0.937	0.931
CG2	0.829	0.91	0.921
CG3	0.746	0.839	0.876
CG4	0.869	0.836	0.792
CG5	0.919	0.893	0.859
CG6	0.94	0.926	0.902
CG7	0.768	0.854	0.869
CG8	0.879	0.9	0.881
CG9	0.778	0.862	0.866
CG10	0.93	0.895	0.851
D1	0.729	0.818	0.846
D2	0.761	0.834	0.868
D3	0.922	0.919	0.912
D4	0.863	0.889	0.908
D5	0.947	0.945	0.946
D6	0.862	0.89	0.898
D7	0.802	0.875	0.912
D8	0.809	0.868	0.896
D9	0.882	0.909	0.915
D10	0.947	0.933	0.912
D11	0.899	0.93	0.95
D12	0.863	0.913	0.926

Source: The results were estimated by the researcher using questionnaires.

Note: LS = Leverage Structure; CG = Corporate Governance; D = Determinant.

**Table 9:** Path coefficients and hypothesis testing.

No	Hypothesis	Coefficient	Std Error	t-value	P-value	$R^{2}$	Decision
1	CG > LSD	1.302	0.115	11.347	0	0.947	Supported
2	D > LSD	-0.336	0.115	2.913	0.002		Supported

Source: The results were estimated by the researcher using questionnaires.

Note that CG = Corporate Governance; D = Determinants of capital structure Decision; LDS = Leverage Structure Decisions.

The coefficient of determination indicates a good fitness for the model, as the model exhibits a strong explanatory power to link between the independent variables and dependent variable. The value 0.947 reveals that totally 95% of financial leverage structure decisions are affected by the corporate governance and determinants. Therefore, the association between corporate governance, determinants and financial leverage decisions firmly established.

## 5 Concluding remarks

The paper aims to examine the perceptions of managers and investors on financial leverage structure decisions in Bangladesh. The study reveals that corporate governance and determinants are the principal significant factors for assessing financial leverage decisions. Corporate governance is positively associated with financial leverage decisions, means that the rising trend of corporate governance quality can enhance firms' creditworthiness in the money and capital markets, as it facilitates the addition of more debt into the leverage structure. The firms, which hold corporate Governance quality will have more access to take outside borrowings, as where firms strictly follow good principles, auditing and control, management skills and monitoring as well, resulting to developing an image over the stock market. As an outcome of corporate governance quality, the board is diversified by professional, managerial, board independence, and dispersed shareholdings followed by developed countries. Such firms severely maintained corporate ethics and principles, which is useful to the firms for easily managing the external financing (Zhou et al., 2021). Contrary to such corporate governance, board was not formed by diversified shareholdings and pattern of shareholdings are different from developed countries that made a restriction to access the stock market for outside borrowings. Such evidence was found in developing counties like Bangladesh, where firms were formed by few shareholdings from government backed and some families, resulting to limited access to stock market, because that firms had lost confidence level of investors over the stock market. Recently firms are very hard to raise the funds from stock market because more than one decade, the stock market was collapsed, as there was no corporate governance (Uddin et al., 2019; Rahman, and Rana, 2018; Rashid, and Johara, 2018). Yet, Kumar and Singh (2013) and Lee et al. (2008) find a positive and significant association between corporate governance and outside borrowing. Although the findings didn't cope with study results of Bansal and Sharma (2016).Results also reveal a negative and significant relationship between determinants and leverage structure decisions, and this relationship implies that when determinants such as profitability, firm size, growth rate, tangibility, and investment opportunity rise, the external borrowing had declined in the leverage structure decisions. Such findings are not expected as suggested by Siddiqui (2012); Jahan (2014). A firm holds less fixed assets or profitable is generally less credit worthiness in the stock market because of having less mortgage, but we find inverse situation in Bangladesh(Rahman, and Rana, 2018; Rashid, and Johara, 2018). Negative signs mainly exist due to government intervention and politics in the banks and stock markets, regardless of other factors. Hossain and Ali (2012) pointed out a negative and significant relationship between determinants and leverage decision. These results neglect the trade-off, agency cost and market timing theory, because for over a decade, the banks and stock markets have been controlled by only few families and directors, who exploit political links to procure funds. Stulz (1990) and Morellec (2004) indicated that the agency cost was an essential factor in implementing the leverage structure decision. Therefore, a negative and significant relationship between the determinants and financial leverage decisions are observed.

## 6 Policy implications

The policy implications should be directed to the following areas: (i) most companies are directed by few families and politicians lacking the required professional know-how. The current ownership structure has promoted an ill-financial policy of firms, limiting the professionalism in management. Therefore, the ownership structure of the Bangladeshi firm should be reformed by dispersing stockholders and amending the current company law in Bangladesh. The amending authorities are government or its agency Bangladesh Security and Exchange Commission (BSEC).(ii). Financial Leverage structure decisions of Bangladeshi firms require a proper building from the pertinent determinants of the capital structure; otherwise, firms may collapse or default earlier than required. The creditworthiness and faith in firms will be restored when firms rationally acquire the leverage structure by trading off their risks and returns. Therefore, the financial managers of firms should build an optimal debt policy basing the proper determinants to cope with risk-return relationship. (iii) The firms should comply with corporate ethics, rules, regulations and financial policies to ensure corporate governance in Bangladesh. Corporate governance enhances the public's confidence and creditworthiness of the firms, and reduces agency problems.

## 7 Limitations

The study needs to extend for some limitations (i) reasons and remedies of political control on corporate leverage decisions in Bangladesh might be investigated to discover the ways to improve the quality of corporate decisions. (ii). Professional Ethics of corporate decision makers on Board meetings are considered high standard to make an efficient and fair decision for which we should capture the further study (iii). Stock market efficiency and corporate debt policy are highly correlated, which is not captured by our study.

## Declarations

### Author contribution statement

Mohammad Nazim Uddin: Conceived and designed the experiments; Performed the experiments; Analyzed and interpreted the data; Contributed reagents, materials, analysis tools or data; Wrote the paper.

### Funding statement

This research did not receive any specific grant from funding agencies in the public, commercial, or not-for-profit sectors.

### Data availability statement

Data will be made available on request.

### Declaration of interests statement

The authors declare no conflict of interest.

### Additional information

No additional information is available for this paper.

## Figures and Tables

**Table:** 

Sr.No.	Company Name	Sr.No.	Company Name
1.	Square Textile Limited	34.	Gemini Sea Food Limited.
2.	Apex Spinning & knitting	35.	Fu-Wang foods Limited.
3.	Delta spinners Limited	36.	National Tea Co Limited.
4.	Rahim Textile Mills Limited.	37.	Information Svs.Net
5.	Saiham Textile Mills Limited.	38.	Eastern Cables Limited.
6.	Sonargaon textiles Limited.	39.	Quasem Dry Cell Limited.
7.	Hr Textile Mills Limited.	40.	National Tubes Limited.
8.	Mithun knitting & Dyeing	41.	Bangladesh Lamps Limited.
9.	Anlima Yarn & Dyeing	42.	Atlas Bangladesh Limited.
10.	Stylecraft	43.	Aftab Automobiles Limited.
11.	Prime Textile Limited.	44.	Singer Bangladesh Limited.
12	Apex Tannery Limited.	45.	Rampur Foundry Limited.
13	Apex Footwear Limited.	46.	National polymer Limited.
14	Eastern Lubricants Limited.	47.	Monno Ceramic Limited.
15	Linde Bangladesh	48.	Standard Ceramic Limited.
16	Padma oil Limited.	49.	Fu-Wang Ceramic Limited.
17	Bangladesh Autocars Limited.	50.	Confidence Cement Limited.
18	Agricultural Marketing Limited.	51.	Meghna Cement Mills Limited.
19	Ibn Sina Limited.	52.	Heidelberg Limited.
20	Libra pharmaceuticals	53.	Aramit Cement Limited.
21	ACI Limited.	54.	Intech online Limited.
22	Renata Limited	55.	BD.com Online Limited.
23	Ambee Pharma Limited.	56.	Beximco Synthetics Limited.
24	Keya Cosmetic Limited.	57.	Eastern Housing Limited.
25	Beximco Pharma Limited.	58.	Samorita Hospital Limited.
26	Square Pharma Limited	59.	GQ Ball Pen Industries Limited.
27	Reckitt Benckiser Limited.	60.	Sino Bangla Industries Limited.
28	Beximco Synthetics	61.	Miracle industries Limited.
29	Kohinoor Chemical Limited.	62.	Usmania Glass Sheet Limited.
30	British American tobacco Bangladesh	63.	Aramit Limited.
31	Apex Foods Limited.		
32	Olympic Industries Limited.		
33	Bangas Limited.		

**Table:** 

Name:				
Age structure:	(a).26–35 years	(b).36-45	(c). 46-55	(d).56–65.
Sex:	(a). Male		(b).Female	
Education:	(a). Graduation		(b)Post Graduation	(c). CA/CMA/FCA
Designation:	Financial Manager or CFO		(b).Chief executive officer or MD	(iii). Board member, or investors
Company’ Name				
Experience	(a). 5–10 (b). 11-15		(c).16–20. (D). 21-25	(e).26–30 Years.

**Table:** 

SL.	Participants in Leverage decisions	Response	
		Yes	No.
1	Board of Directors		
2.	Chief Financial officer/Financial Managers		
3.	Chief Executive officer/Managing director		

**Table:** 

Q.N.	Determinants/Variables	5	4	3	2	1
$D_{1}$	Profitability results in leverage decision					
$D_{2}$	Growth opportunity influences the leverage decision					
$D_{3}$	Tangibility has an impact on leverage structure					
$D_{4}$	Changes in market interest rates influence leverage decision					
$D_{5}$	Corporate tax rates directly involve leverage decision					
$D_{6}$	Business risks influence leverage decisions					
$D_{7}$	Agency cost is related to leverage decision					
$D_{8}$	Liquidity has an impact on leverage decision					
$D_{9}$	Investment opportunities directly influence leverage decision					
$D_{10}$	The firm size can influence leverage decision					
$D_{11}$	The nature of industry influences leverage decision					
$D_{12}$	Stock price volatility influences leverage decision					

Note: Strongly agree = 5; Agree = 4, neutral = 3; Disagree = 2; strongly disagree = 1.

**Table:** 

Q.N.	Determinants/Variables	5	4	3	2	1
$CG_{1}$	The rights of shareholders are recognized by your firm					
$CG_{2}$	Board Size and composition have an influence on leverage decisions					
$CG_{3}$	Corporate laws and regulations are observed by your firm					
$CG_{4}$	Independent directors are on board (at least 10%)					
$CG_{5}$	The rights of stakeholders are protected by law.					
$CG_{6}$	Financial reports are timely prepared and delivered to stakeholders					
$CG_{7}$	Firms can freely appoint auditors					
$CG_{8}$	Your firm provides fairly, timely and cost effective information					
$CG_{9}$	Annual general meeting(AGM) is regularly held					
$CG_{10}$	There is female board member participation on the board.					

Note: Strongly agree = 5; Agree = 4, neutral = 3; Disagree = 2; strongly disagree = 1.

**Table:** 

Q.N.	Sources of financing or capital mix	5	4	3	2	1
$LS_{1}$	Issuing common stock/external equity financing					
$LS_{2}$	Issuing bond/debenture/Long-term loan					
$LS_{3}$	Issuing preferred stock (redeemable/Perpetual)					
$LS_{4}$	Retained Earnings/Internal equity financing					

Note: Strongly agree = 5; Agree = 4, neutral = 3; Disagree = 2; strongly disagree = 1.

## Appendix 1

List of Sample Companies.

## Appendix 2

Leverage Structure Decisions in Bangladesh: Managers and Investors’ View.

1. Demographical information of Respondents.

2. Who become (s) responsible for leverage structure Decisions in your firm?

3. Specify the determinants that influence the leverage structure decision in Bangladesh.

4. Identify the factors of corporate governance that influence the leverage structure Decisions in Bangladesh.

5. Determine the financing mix for leverage structure decisions from the following sources:
